# Correction: Effectiveness of blocking primers and a peptide nucleic acid (PNA) clamp for 18S metabarcoding dietary analysis of herbivorous fish

**DOI:** 10.1371/journal.pone.0333594

**Published:** 2025-09-30

**Authors:** The PLOS One Staff

In Table 1, underlines of tag sequences of the primers and units of melting temperature: Tm^*3^ (°C), were missing. Please see the correct Table 1 here.

**Table 1:** List of the primers and the PNA clamp used in this study for metabarcoding.

Name	Primer/PNA	Direction	Sequence (5’–3’)^*1^	Binding site*^2^	Length	Tm^*3^(°C)	Reference
V8f	Primer	Forward	ATAACAGGTCTGTGATGCCCT	1,487–1,507	21	58.8	Bradley et al. (2016)
18SV9R	Primer	Reverse	CCTTGTTACGACTTYTMCTTCCTCTA	1,814–1,839	26	58.6-61.5	This study
V8f_tag	Primer	Forward	TCGTCGGCAGCGTCAGATGTGTATAAGAGACAGATAACAGGTCTGTGATGCCCT	1,487–1,507	21	58.8	Bradley et al. (2016)
18SV9R_tag	Primer	Reverse	GTCTCGTGGGCTCGGAGATGTGTATAAGAGACAGCCTTGTTACGACTTYTMCTTCCTCTA	1,814–1,839	26	58.6-61.5	This study
BlockFish5	Primer	Reverse	CTCTAGATAGTCAAGTTTGATCG	1,796–1,818	23	54.2	This study
BlockFish10	Primer	Reverse	ACTTCCTCTAGATAGTCAAGTTTGATCG	1,796–1,823	28	60.4	This study
BlockFish_long6	Primer	Reverse	CCTCTAGATAGTCAAGTTTGATCGTCTTCTCGGC	1,786–1,819	34	67.4	This study
BlockFishPNA	PNA	Reverse	CTCTAGATAGTCAAGTTTGATCG	1,796–1,818	23	77.5	This study

*1: Underline: overhang adapter sequence.

*2: Base position based on the 18S rDNA sequence of Verasper variegatus (Acc. No. EF126043).

*3: Tm calculated using the nearest neighbor method.

In the Introduction, there is an error in the fifth sentence of the fourth paragraph. The correct sentence is: However, whether blocking primers or PNA clamps are suitable as blocker for metabarcoding fish prey organisms has not been examined.

The publisher apologizes for the errors.
