# Supplementary figures and images for: Effectiveness of blocking primers and a peptide nucleic acid (PNA) clamp for 18S metabarcoding dietary analysis of herbivorous fish
Source: PLoS One. 2022 Apr 20;17(4):e0266268. doi: 10.1371/journal.pone.0266268 (PMC9020718; doi:10.1371/journal.pone.0266268)

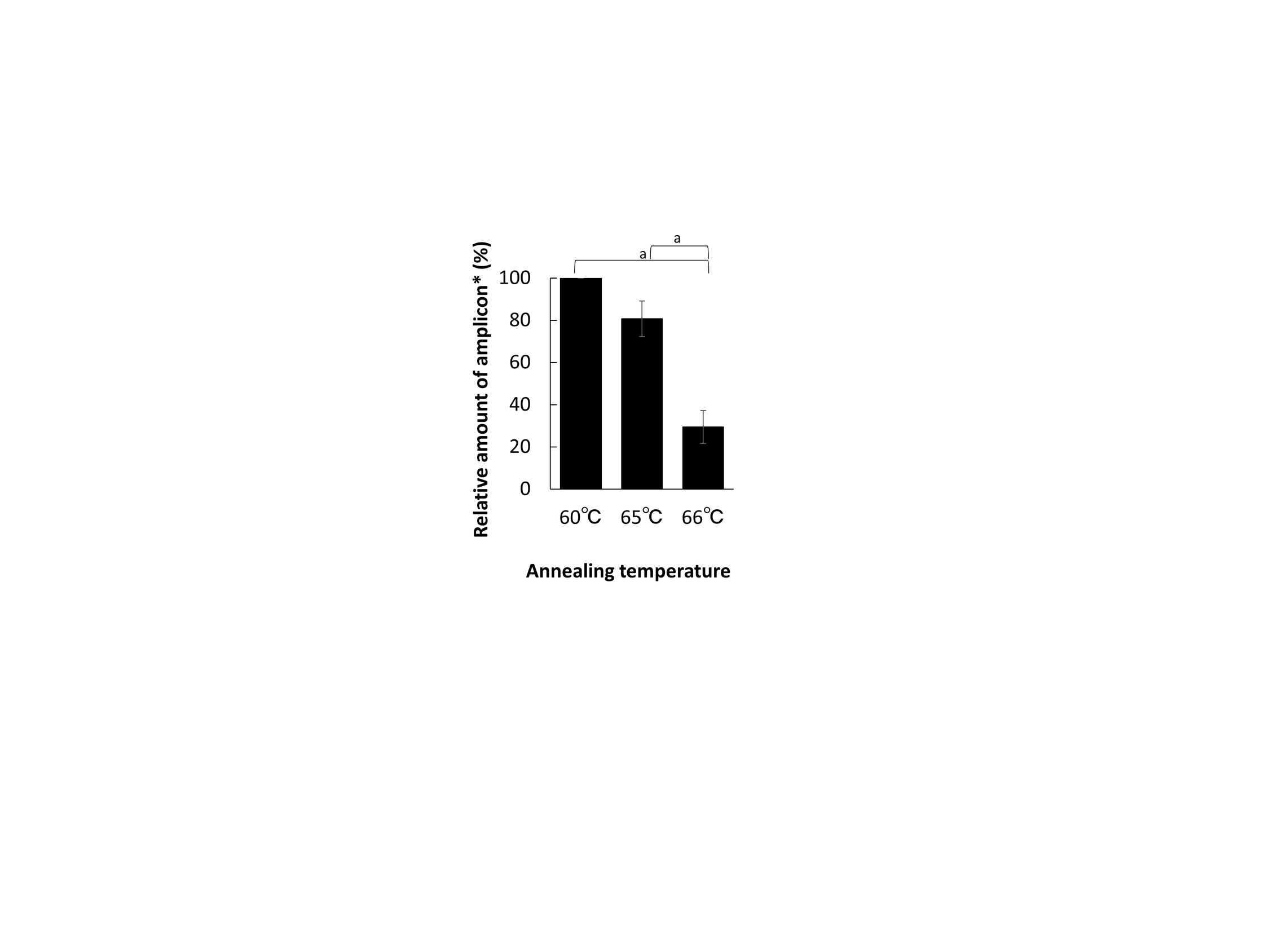

Supplement: S1 Fig — a: Tukey-Kramer test: p<0.01. *: Relative amount of amplicon when the amount of amplicon at 60°C was regarded as 100%. (TIF) [file pone.0266268.s001.tif]

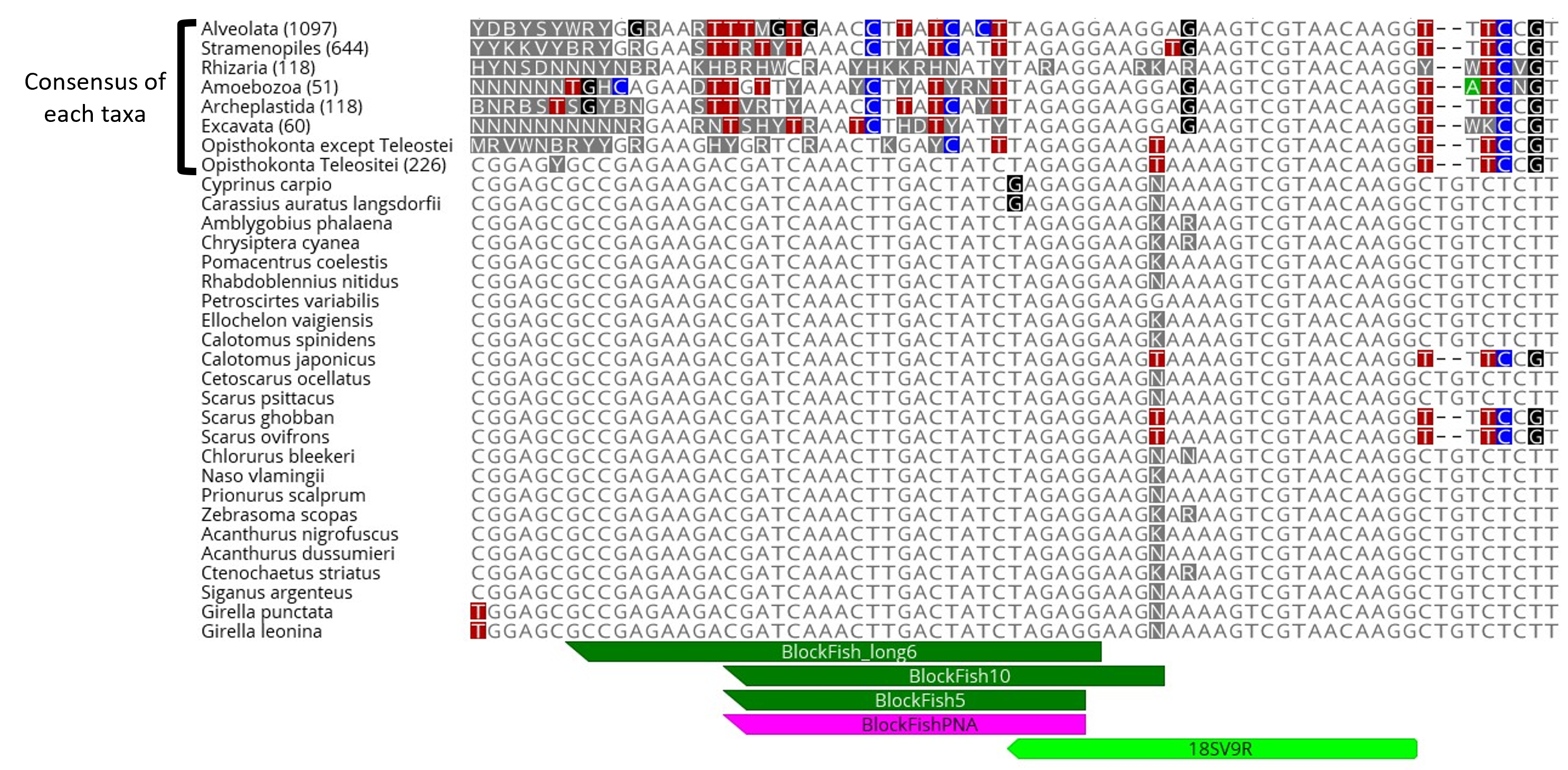

Supplement: S2 Fig — (TIF) [file pone.0266268.s002.tif]
