# Supplementary material for: Effectiveness of blocking primers and a peptide nucleic acid (PNA) clamp for 18S metabarcoding dietary analysis of herbivorous fish
Source: PLoS One. 2022 Apr 20;17(4):e0266268. doi: 10.1371/journal.pone.0266268 (PMC9020718; doi:10.1371/journal.pone.0266268)
Supplement: S1 Table — (DOCX) [file pone.0266268.s003.docx]

S1 Table. List of herbivorous fish used in this study.

| **Order** | **Family** | **Species** | **Date** | **Location** | **Provider** | **Method of collection** | **Acc. No. of 18S rDNA** |
| --- | --- | --- | --- | --- | --- | --- | --- |
|  |  |  |  |  |  |  |  |
| Cypriniformes | Cyprinidae | *Cyprinus carpio* | ND^*1^ | ND^*1^ | Pet shop^*2^ | ND^*1^ | LC639918 |
|  |  | *Carassius auratus langsdorfii* | ND^*1^ | ND^*1^ | Pet shop^*2^ | ND^*1^ | LC639919 |
| Gobiiformes | Gobiidae | *Amblygobius phalaena* | 2018.5 | Laguindingan, Mindanao Island, Philippines (8.37, 124.27) | Y. Nakamura | Seine net | LC639922 |
| Series Ovalentaria | Pomacentridae | *Chrysiptera cyanea* | ND^*1^ | ND^*1^ | Pet shop^*2^ | ND^*1^ | LC639924 |
|  |  | *Pomacentrus coelestis* | 2018.10 | Ikenoura, Susaki City, Kochi, Japan (33.24, 133.24) | Y. Nakamura | Hand net | LC639925 |
|  | Blenniidae | *Rhabdoblennius nitidus* | ND^*1^ | ND^*1^ | Pet shop^*2^ | ND^*1^ | LC639926 |
|  |  | *Petroscirtes variabilis* | 2018.5 | Laguindingan, Mindanao Island, Philippines (8.37, 124.27) | Y. Nakamura | Seine net | LC639927 |
|  | Mugilidae | *Ellochelon vaigiensis* | 2018.5 | Laguindingan, Mindanao Island, Philippines (8.37, 124.27) | Y. Nakamura | Seine net | LC639928 |
| Series Eupercaria | Scaridae | *Calotomus spinidens* | 2018.5 | Laguindingan, Mindanao Island, Philippines (8.37, 124.27) | Y. Nakamura | Seine net | LC639929 |
|  |  | *Calotomus japonicus* | 2012.11.5 | Kutsu, Susaki City, Kochi, Japan (33.396, 133.340) | Z. Imoto | Spear fishing | LC639930 |
|  |  | *Cetoscarus ocellatus* | ND^*1^ | ND^*1^ | Pet shop^*2^ | ND^*1^ | LC639931 |
|  |  | *Scarus psittacus* | 2018.12 | Laguindingan, Mindanao Island, Philippines (8.37, 124.27) | Y. Nakamura | Seine net | LC639932 |
|  |  | *Scarus ovifrons* | 2015.7.13 | Muroto Misaki, Muroto City, Kochi, Japan (33.266, 134.160) | K. Yamagata | Bait fishing | LC639933 |
|  |  | *Scarus ghobban* | 2012.3.27 | Kutsu, Susaki City, Kochi, Japan (33.396, 133.340) | Z. Imoto | Spear fishing | LC639934 |
|  |  | *Chlorurus bleekeri* | 2018.12 | Laguindingan, Mindanao Island, Philippines (8.37, 124.27) | Y. Nakamura | Seine net | LC639935 |
|  | Acanthuridae | *Naso vlamingii* | 2018.12 | Laguindingan, Mindanao Island, Philippines (8.37, 124.27) | Y. Nakamura | Seine net | LC639936 |
|  |  | *Prionurus scalprum* | 2018.10 | Ikenoura, Susaki City, Kochi, Japan (33.24, 133.24) | Y. Nakamura | Spear fishing | LC639937 |
|  |  | *Zebrasoma scopas* | ND^*1^ | ND^*1^ | Pet shop^*2^ | ND^*1^ | LC639939 |
|  |  | *Acanthurus nigrofuscus* | 2018.12 | Laguindingan, Mindanao Island, Philippines (8.37, 124.27) | Y. Nakamura | Seine net | LC639938 |
|  |  | *Ctenochaetus striatus* | 2018.12 | Laguindingan, Mindanao Island, Philippines (8.37, 124.27) | Y. Nakamura | Seine net | LC639941 |
|  | Siganidae | *Siganus argenteus* | 2018.12 | Laguindingan, Mindanao Island, Philippines (8.37, 124.27) | Y. Nakamura | Seine net | LC639942 |
|  | Kyphosidae | *Girella punctata* | 2018.10 | Ikenoura, Susaki City, Kochi, Japan (33.24, 133.24) | Y. Nakamura | Spear fishing | LC639943 |
|  |  | *Girella leonina* | 2018.10 | Ikenoura, Susaki City, Kochi, Japan (33.24, 133.24) | Y. Nakamura | Spear fishing | LC639944 |
|  |  |  |  |  |  |  |  |

^*1^: No data

^*2^: Pet shop located in Kochi City, Kochi, Japan
