# Supplementary material for: Effectiveness of blocking primers and a peptide nucleic acid (PNA) clamp for 18S metabarcoding dietary analysis of herbivorous fish
Source: PLoS One. 2022 Apr 20;17(4):e0266268. doi: 10.1371/journal.pone.0266268 (PMC9020718; doi:10.1371/journal.pone.0266268)
Supplement: S2 Table — (DOCX) [file pone.0266268.s004.docx]

S2 Table. Number of 18S rDNA sequences of each taxon taken from NCBI for designing the universal reverse primer and fish (Teleostei) blockers.

| **Taxon** | **Number of sequences** |
| --- | --- |
|  |  |
|  |  |
| Alveolata | 1097 |
| Stramenopiles | 644 |
| Rhizaria | 118 |
| Amoebozoa | 51 |
| Archaeplastida | 118 |
| Excavata | 60 |
| Opisthokonta | 625 |
| Teleostei (Opisthokonta) | 226 |
|  |  |
| Total | 2713 |
|  |  |
