# Supplementary material for: Effectiveness of blocking primers and a peptide nucleic acid (PNA) clamp for 18S metabarcoding dietary analysis of herbivorous fish
Source: PLoS One. 2022 Apr 20;17(4):e0266268. doi: 10.1371/journal.pone.0266268 (PMC9020718; doi:10.1371/journal.pone.0266268)
Supplement: S3 Table — (DOCX) [file pone.0266268.s005.docx]

S3 Table. Number of mismatches between the sequence of each blocker and the 18S rDNA sequence of each taxon in the region of the blocker.

| **Organism** | **Number of mismatches** | |
| --- | --- | --- |
|  |  |  |
|  | **BlockFish_long6** | **BlockFishPNA** |
|  |  |  |
|  |  |  |
| *Scarus ovifrons* | 0 | 0 |
| *Zonaria diesingiana* | 5 | 5 |
| *Gelidium* sp. | 8 | 6 |
| *Ulva reticulata* | 9 | 6 |
| *Symbiodinium* sp. | 13 | 8 |
| *Pagurus filholi* | 15 | 11 |
| Nereididae sp. | 17 | 12 |
| Euphylliidae sp. | 18 | 13 |
|  |  |  |
