# Supplementary material for: Effectiveness of blocking primers and a peptide nucleic acid (PNA) clamp for 18S metabarcoding dietary analysis of herbivorous fish
Source: PLoS One. 2022 Apr 20;17(4):e0266268. doi: 10.1371/journal.pone.0266268 (PMC9020718; doi:10.1371/journal.pone.0266268)
Supplement: Attachment — Submitted filename: Response to Reviewers combined 021922.docx [file pone.0266268.s006.docx]

**Dear Reviewers,**

We are grateful for your valuable comments and suggestions that have helped to improve our manuscript (MS), entitled "Effectiveness of the blocking primer and PNA clamp against for 18S metabarcoding dietary analysis of herbivorous fishes" by Chiho Homma, Daiki Inokuchi, Yohei Nakamura, Wilfredo H. Uy, Kouhei Ohnishi, Haruo Yamaguchi, Masao Adachi as an Original Research Article for publication in PLoS One. We have considered the comments and suggestions in the revised version of our MS. Responses to your comments are presented below.

**Responses to Reviewer #1’s Comments:**

**Comment 1:** The authors have stated the limitations of previously used methods, but the limitations of DNA barcoding accompanied with molecular cloning as a method could be elaborated a little more to consolidate their point of the need for a new method (inclusion of PNA clamp in this case during amplification) because the point that it is laborious and time consuming (line 44-47) could be applicable to most of the processes described and is general in my opinion. As DNA barcoding has been previously used for determining the prey organism of *Siganus fuscescens* and *Scarus ovifrons*, I think it would be better to specify the limitations from that experiment as well to strengthen the claims made by the author.

**Response 1:** Thank you for your suggestion. Considering your suggestion, we described the limitations from the DNA barcoding in the revised Introduction of our manuscript (revised MS Page 3, Lines 45 – 49) as described below:

‘The DNA barcoding [18,19] accompanied by single-stranded conformational polymorphism (SSCP) analysis or molecular cloning of PCR products circumvents the problem described above, but these approaches need DNA sequencing of many PCR products from the SSCP analysis or DNA sequencing of many clones derived from the PCR products, which are laborious and time-consuming [20].’

**Comment 2:** Regarding the table no. 4. For *langsdorfii,* no observations are included in the table and the genus name has not been provided, please address to that as it has been mentioned in line no. 325-327 of the manuscript in relation to a 100% suppression by BlockFish_long7

**Response 2**: *Carassius auratus langsdorfi* is one species name. In the original manuscript, there were separated into two lines, which made it difficult to understand. Therefore, the species name was shown in a single line in Table 4 in the revised MS.

**Comment 3:** Also, Since *Naso vlamingii* does not seem to produce amplicons without blockers at 65 and at 60 degrees Celsius, and produces only 0-50% of relative amount of amplicons, was it tested at lower annealing temperatures than 60 degrees to see if more amplicons are being produced for *Naso vlamingii* without the blocker. Was there any limitation that did not allow more observations for the said species such as the sample size or availability.

**Response 3**: In Table 4, we showed effectiveness of BlockFish_long6 and BlockFishPNA to various fishes. No amplicon of *Naso vlamingii* was obtained at 65℃, but an amplicon of it was obtained at 60°C, although the amount of the amplicon was small (0-50%). We think that it is not necessary to test the effectiveness of blockers at lower annealing temperatures than 60 degrees, because non-specific amplification may occur if the annealing temperature is lowered below this temperature (60 degrees) which is about Tm of the universal primer. So we did not test temperatures less than 60℃.

**Comment 4**: Please include the page numbers and make sure to review the submission guidelines for the journal to check the formatting of the manuscript.

**Response 4:** Following your suggestion, we have added the page numbers in the revised MS. Thank you.

**Comment 5**: A general question for someone not a specialist in this field, This is more of a personal curiosity regarding the use of V9 and V8f region, Is there a possibility to use any more regions on 18S rDNA or an inclusion of more regions of 18S rDNA as biomarker, if yes would that be beneficial or the results would be similar in preparing the blockers and PNA clamp?

**Response 5**: We think that it is possible to use regions other than 18S rDNA V8-V9 used in this study, such as mitochondrial CO1 as barcode regions. In the cases, it is important to consider a use of a PNA clamp or a use of a blocking primer having higher Tm than that of a universal PCR primer. In addition, it is desirable to optimize the concentration of the blocker and the annealing temperature as investigated in this study.

**Responses to Reviewer #2’s Comments:**

Reviewer #2: The manuscript shows how efficiently the gut microbiology of herbivorous fish can be investigated by focusing on 18S rDNA of only prey organisms and blocking the DNA content of fish. The study was novel as the prey content has not been identified in herbivorous fish. The manuscript is well written. Would suggest minor revision. The changes required are:

**Comment 1**: The details of designing and selecting the primers were not clear in the manuscript. If added in supplementary, I would suggest to add that in the materials and methods section where the primer designing has been mentioned. Also mention from which organism/s each primers has been designed.

 **Response 1**: We created a multiple alignment using the 226 Teleostei sequences, 2,713 sequences of various eukaryotic organisms other than Telostei from NCBI (Table S2), and the 24 fish sequences determined in this study. In the revised manuscript, we showed the alignment in Fig. S2 described below which shows the annealing sites of the newly designed universal reverse primer 18SV9R, the blocking primers and the PNA clamp used in this study.

**Comment 2**: I feel metabarcoding results should also be properly mentioned. The prey organisms mentioned in Table 2 were only used for conducting mock tests? Incorporate the metabarcode analysis of prey organisms (the list of organisms or classes of organisms that are observed in fish by PCR blocking technique).

**Response 2**: We are sorry that we have not yet applied the metabarcode analysis using the blockers to analysis of prey organisms of herbivorous fishes. In future, we would like to report prey organisms of various herbivorous fishes analyzed by a metabarcode analysis using the blockers reported in this study.

**Responses to Reviewer #3’s Comments:**

**Comment 1:** BlockFish_long7 has only 6 nucleotide overlap with the 18SV9R which does match with the text in line 119.

**Response 1:** Considering your comment, we have changed the name of the blocker from BlockFish_long7 to BlockFish_long6 in the text as well as all figures and tables. Thank you.

**Comment 2**: In the Table 2 labels of columns Mock 1, Mock 2 and Mock 3 does not give the idea about what the numbers given in the columns represent (in line 236 mentioned but it should be clearly labeled in table also)

**Response 2:** Considering your comment. we have explained the meaning of the number in the footnote of the table as shown below:

‘Number in column showed a ratio of each organism in the mock community samples.’

**References**

We have inserted spaces before volume numbers and deleted spaces before page numbers.

We have added information about article numbers or a volume number of some references (Ref.: 5, 19, 26, 27, 29, 31, 32, 36, 37).

We have changed the doi of Ref: 44.
